# The Gallipot Method—A Point of Technique for Safe and Easy Staple Removal From Split-Thickness Skin Grafts

**Published:** 2018-11-19

**Authors:** Stephen R. Ali, Jonathon Pleat

**Affiliations:** ^a^Department of Plastic and Reconstructive Surgery, Norfolk and Norwich University Hospital, Norwich, United Kingdom; ^b^Department of Plastic, Reconstructive and Burns Surgery, North Bristol NHS Trust, Southmead Hospital, Southmead Road, Bristol, United Kingdom

**Keywords:** Split-thickness skin graft, staples, removal

Dear Sir,

Split-thickness skin grafts are commonly used to resurface wounds in plastic surgery. Surgical staples are a quick way to secure the graft to the wound bed and facilitate take. Despite these advantages, the process of removing staples can cause inadvertent trauma to the newly inset, fragile graft. We describe a novel method where a 4-mm biopsy punch is used to cut 3 holes in series at the bottom of a polypropylene gallipot placed upon a hard surface (Fig 1). This permits staple removal directly into the gallipot through the holes. Aperture width can be widened if necessary by repeat punching on the perpendicular axis. The flat base of the gallipot simultaneously applies counter-pressure upon the bed to eliminate graft tenting as staples are removed with mosquito forceps or equivalent instruments (Fig 2). All sharps are confined to the gallipot, reducing risk of injury by eliminating transit across the surgical field. The gallipot is then advanced to remove staples in sequence. The technique does require evenly spaced staples for direct access through the gallipot. This simple and inexpensive method is used in both the dressings clinic and theatre settings at our institutions to achieve atraumatic staple removal. It can be reproduced in any surgeon's practice, takes minimal time to implement, and utilizes equipment and materials readily available in most operating theatres.

## Figures and Tables

**Figure 1:**
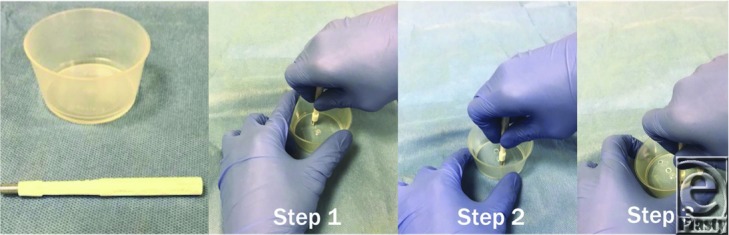
Preparing the gallipot to be used. Steps 1 to 3 can be repeated so that the whole staple is accessible along its length by having multiple holes lined up together.

**Figure 2:**
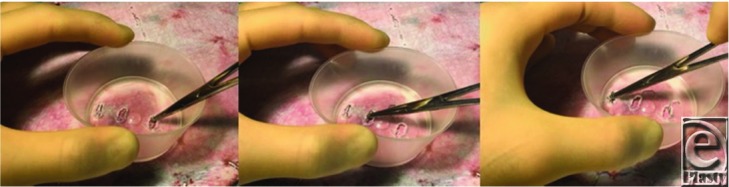
The gallipot technique in use. Access and removal of surgical staples through the gallipot in sequence. Aperture width has been widened by repeating steps 1 to 3 in Figure 1.

